# Supplementary material for: Impact of staffing model conversion from a mandatory critical care consultation model to a closed unit model in the medical intensive care unit
Source: PLoS One. 2021 Oct 27;16(10):e0259092. doi: 10.1371/journal.pone.0259092 (PMC8550369; doi:10.1371/journal.pone.0259092)
Supplement: Attachment — Submitted filename: Closed unit Revision response Final (PLoS One) 0925.docx [file pone.0259092.s002.docx]

Dear Dr. Emily Chenette,

Editor-in-Chief of *PLoS One*

We wish to thank the editor and reviewers for their response to our work and their constructive comments. We have addressed the concerns that were raised through further editing of the manuscript and believe it has significantly improved as a consequence of this process. We have highlighted all changes in the revised manuscript and have addressed all referees’ comments in the point-by-point responses below. We greatly appreciate the opportunity to submit this revised review manuscript and hope it is suitable for publication in *PLoS One*.

4. Have the authors made all data underlying the findings in their manuscript fully available?

Reviewer #1: No

**🡪 We apologize that the data cannot be shared publicly due to potentially identifying or sensitive information. Requests for data access may be sent to Seoul National University Institutional Data Access / Ethics Committee (contact via Tel: 82-2-2072-0694) for researchers who meet the criteria for access to confidential data.**

6. Review Comments to the Author

Reviewer #1: I would like to thank the authors for addressing the reviewers' comments. In my opinion, a final check for linguistic flaws (e. g. "twice as more patients" in the new paragraph of the limitations section schould be changed to "twice as many") ought to be the last step before acceptance.

**🡪 We thank the reviewer for this valuable comment. We performed grammatical corrections on the revised text, and as a result, the following sentences were corrected.**

**Introduction 27-30th line: Corrected “The aim of this study was to compare closed unit model to mandatory critical care consultation model and to evaluate the superiority of the closed unit model in the outcomes of critically ill Asian patients.” to “This study aimed to compare the closed unit model to the mandatory critical care consultation model and evaluate the superiority of the closed unit model in terms of outcomes in critically ill Asian patients.”**

**Result 1st-2nd line: Corrected “1,076 patients in mandatory critical consultation model group and 581 patients in closed unit model group” to “1,076 and 581 patients in the mandatory critical consultation model group and the closed unit model group, respecively”**

**Result 3rd-5th line: Corrected “89 [8.3%] patients in mandatory critical care consultation model group and 42 [7.2%] patients in closed unit model group” to “89 [8.3%] and 42 [7.2%] patients in mandatory critical care consultation model group and the closed unit model group, respectively”**

**Discussion 21th line: Corrected “Despite patients having higher APACHE II scores at admission” to “Although the patients had higher APACHE II scores at admission”**

**Discussion 41-42th line: Corrected “It is impossible to completely exclude the possibility that outcome changes may have been from advances in medicine rather than changes in staffing models” to “Further, the outcome changes might have been due to advances in medicine rather than changes in staffing models”**

**Discussion 49-54th line: Corrected “Third, there are almost twice as more patients in the mandatory consultation model compared to the closed unit model. This was due to differences in the duration of the before and after staffing models. While equal-sized groups have maximal statistical power, we believe that the smaller group of our study (n=539) was still large enough sample to detect clinical significant differences between the two groups” to “Third, the number of patients in the mandatory consultation model group was twice as high as that than in the closed unit model group. This was due to differences in time period before and after changing the staffing model. Although equal-sized groups have maximal statistical power, we believe that the smaller size of the group in our study (n=539) was large enough to detect clinically significant differences between the two groups”.**

Reviewer #2: Thank you for submitting your revisions and addressing all recommendations by reviewers. No further suggestions on my end.

**🡪 Thanks for your thoughtful comment.**
